# Determinant factors of home delivery among women in Northern Ethiopia: a case control study

**DOI:** 10.1186/s12889-017-4159-1

**Published:** 2017-04-04

**Authors:** Resom Tsegay, Alemseged Aregay, Kalayu Kidanu, Mussie Alemayehu, Gebrezigabiher Yohannes

**Affiliations:** 1 Areaya Kahsu Health Science college, Department of Midwifery, Axum, Tigray Ethiopia; 2 Mekelle university, College of health science, School of public health, Mekelle, Tigray Ethiopia; 3 Mekelle university, College of Health Science, Department of Nursing, Mekelle, Tigray Ethiopia; 4 Millennium Health Science college, Department of Nursing, Mekelle, Tigray Ethiopia

**Keywords:** Home delivery, Maternal mortality, Tanqua-Abergele, Tigray, Ethiopia

## Abstract

**Background:**

Maternal mortality remains a major challenge to health systems worldwide. Although most pregnancies and births are uneventful, approximately 15% of all pregnant women develop potentially life-threatening complications. Home delivery in this context can be acutely threatening, particularly in developing countries where emergency care and transportation are less available. This study identifies factors associated with home delivery in Tanqua-Abergele District, Tigray, northern Ethiopia.

**Methods:**

Unmatched case-control study was conducted in April 2014 in Tanqua-Abergele, Tigray, northern Ethiopia. Simple random sampling was employed to select study participants. Data were analyzed using SPSS 20. Multi variable logistic regression analysis was used to identify independent predictors of home delivery.

**Results:**

A total of 275 women (92 cases and 183 controls) participated in the study, giving a response rate of 96.5%. Not owning a radio or television (AOR: 7.2, 95% CI: 2.7–19.3), not pursuing ANC visits at all (AOR: 10.4, 95% CI: 2.9–37.1) orhaving1–3 ANC visits only (AOR: 4.75, 95% CI: 1.69–13.31),poor knowledge of obstetric complications (AOR: 8.7, 95% CI: 2.3–32.9) and walking time greater than two hours to the nearest health center (AOR: 5.1, 95% CI: 1.2–20.7) were strong predictors of home delivery.

**Conclusion:**

Unable to meet the minimum requirement WHO of ANC service had a potential to give birth at home. Investing in infrastructure will contribute to improving maternal health. Having a different source of media (radio or television) could have a role in increasing the institutional delivery. Policy makers and other concerned bodies should give due attention to the fulfillment of infrastructure and educate women on the importance of institutional delivery.

## Background

Maternal mortality remains a major challenge to health systems worldwide. Globally, there were 287,000 maternal deaths in 2010. Of the 287,000 maternal deaths worldwide, 85% occurred in Sub-Saharan Africa (SSA) and Southern Asia [1]. The maternal mortality rate (MMR) in Ethiopia is high (676/100,000 live births), despite the recognition of maternal mortality as a major public health issue [2]. Skilled attendants assist in more than 99% of births in developed countries compared with 62% in developing countries [3]. In Ethiopia, the situation is even worse – nine in ten women give birth at home [2]. Based on Tigray Regional Health Bureau’s annual profile 2013, home deliveries in the region and Tanqua-Abergele District were 43.4% and 62.8%, respectively [4]. Home deliveries are bound to be un-hygienic, unsupervised and, when intervention is required, it is usually late [3].

One factor associated with maternal and neonatal mortality is home delivery. These deliveries are largely unplanned, accidental and supported by unskilled health professionals, if at all. The home environment as a place of delivery in developing countries is shown to unsafe and may have adverse neonatal and maternal outcomes [5]. Interestingly, a large proportion of these maternal deaths could be prevented through timely and appropriate interventions. The presence of skilled delivery service utilization at each birth can significantly reduce maternal morbidity and mortality [6, 7]. Home delivery is associated with young maternal age, low educational attainment, rural residence, low socioeconomic status, high birth order, the absence of ANC services, distance to health facilities and complications during delivery [8–10].In contrast, having good knowledge of obstetric complications can enhance institutional delivery [11]. Still, many studies show that mother’s awareness of danger signs of pregnancy is poor and affected by educational status, occupation-[12], and residential area [13].

During the past fifteen years, the Federal Ministry of Health (FMoH) has built an impressive framework for improving health for all, including maternal and neonatal health. There are strategies for free maternal and child health services. Activities include deploying Health Officers with MSc training in Integrated Emergency Obstetrics and Surgery (IEOS), improving the availability of safe blood and pharmaceutical supplies and a strong referral system. In addition to these activities, Tigray region has undertaken different activities to avert home delivery [4], including establishing a new structure at the community level below Health Extension Workers (HEW). The “Women Development Army” enables women to talk about pregnancy and delivery, making health facilities more friendly to the community, creating forums for pregnant women, the opening of maternity waiting home at health facilities, increasing the number ambulance services and availing of traditional ambulances for early referrals in case of emergencies [4]. Despite these efforts, the rate of home delivery in Tanqua-Abergele District is still high.

## Methods

The study was conducted in Tanqua-Abergele District in northern Ethiopia from April 1 to 30, 2014. Tanqua-Abergele has five health centers and 16 health posts [4]. A community-based unmatched case-control study was used to enroll women who gave birth in the 6 months preceding data collection. Among this group, women who gave birth at home were considered as cases and those who gave birth at health institutions were controls. The total number of pregnant women was 3,360 and there were 331 home and 931 institutional deliveries from July-December 2013(report from Tanqua-AbergeleWoreda Health Office, December 2013).

The following assumptions were made in calculating sample size: 95% confidence level and 80% power. General knowledge about obstetric complications was used as the exposure variable. The proportion of controls with good knowledge was 80% as well as 62.9% of cases [11]. Sample size was calculated using Open Epi version 2.3 with a case to control the ratio of 1:2. After adding 10% non-response, total sample size was 285, including 95 for cases and 190 for controls. Simple random sampling was used to select study participants using the master family index as the sampling frame. Tanqua-Abergele has a total of 20 *kebeles*, the lowest administrative unit in Ethiopia, and all kebeles were included in the study. Sample was allocated proportionally to population across all 20 kebeles based on the number of women who gave birth at home and health institution in the six months preceding the survey.

A semi-structured, pre-tested questionnaire adapted from several studies [2, 11, 14, 15] and covering socio-demographic, health care and obstetric related factors were used. Ten data collectors who had completed Grade 10 and two (2) diploma nurse supervisors were trained for two days. The questionnaire was initially prepared in English and translated to Tigrigna. It was then checked for consistency by back-translation to English by language experts. Pretest was done two weeks before the survey in Deguaa-TembenWoreda, on 14 (5%) of the sample (5cases and 9controls). Based on the pretest, a questionnaire was corrected to ensure clarity, wording, and logic sequence and skip patterns.

### Measurement

Knowledge about danger signs during pregnancy was measured by comparing mean score responses to eight danger signs. Those with scores at or below the mean were classified as having “poor knowledge” while those with scores above the mean were categorized as having “good knowledge” [11].

General knowledge of obstetric complications was measured by calculating composite scores to seven obstetric complications, which were then categorized into” good”, “fair” and “bad “levels of knowledge. Respondents with “good knowledge” included that scoring 75% and above; “moderate knowledge” included those with scores between 50 to 74% and; “poor knowledge” characterized those that scored less than 50% [11].

### Data processing and analysis

Data entry, cleaning, and analysis were done using SPSS version 20. Data was summarized and descriptive statistics were computed for all variables according to type. Frequency, mean and standard deviation were obtained for continuous variables while categorical variables were assessed using frequencies. A binary analysis was used to describe the association between independent and dependent variables. A multivariable logistic regression analysis identified determinants of home delivery at 95% CI and *P*-value < 0.05. The final model was fitted using the Hosmer-Lemeshow Goodness of Fit test. Multicollinearity was checked along with all the analysis.

## Results

### Socio-demographic characteristics of the study participants

A total of 275 respondents (92 cases and 183 controls), participated in the study, giving a response rate of 96.5%. Mean age of cases and controls was 28.6 (SD ± 5.5) and 26.6 (SD ± 5.9) years, respectively, and ranging from 16 to 39 years. Majority of cases were illiterate (68, 73.9%) cases as well as 96 (52.5%) controls. Median monthly family income for cases and controls was $22.50(IQR ± $22.50) and $37.50(IQR ± 400), respectively. Almost all respondents were Orthodox Christian and married (256, 93.1%). Forty-four (47.8%) cases and 89 (48.6%) controls were farmers and housewives by occupation, respectively. Approximately half of cases’ husbands were illiterate. Eighty-three (49.4%) controls’ husbands’ had attended grade one to eight, respectively. Around 75% of cases and 45.4% of controls did not have a radio or television (Table 1).

**Table 1:** Socio-demographic characteristics of study participants in Tanqua-Abergele District, Tigray, north Ethiopia, 2014

Variables	Cases No (%)	Controls No (%)	Chi-square, df, *p*-value
Residence of respondents			
Rural	90(97.8)	138 (75.4)	χ2 = 21.71, df =1
Semi urban	2 (2.2)	45(24.6)	*P*-value = 0.000
Age of respondents			
15–19	3(3.3)	25(13.7)	χ2 = 15.361
20–24	21(22.8)	46(25.1)	df = 4
25–29	24(26.1)	58(31.7)	*p*-value = 0.004
30–34	26(28.3)	24(13.1)	
35–39	18(19.6)	30(16.4)	
monthly income ($)			
< 50	85(92.2)	136(74.3)	χ2 = 12.69
50–99.5	4(4.3)	25(13.7)	df = 2
> =100	3(3.3)	22(12.0)	*p*-value = 0.002
Marital status			
Married	88(95.7)	168 (91.8)	χ2 = 1.41, df = 1
Others^a^	4 (4.3)	15(8.2)	*p*-value = 0.235
Maternal educational level			
Illiterate	68(73.9)	96(52.5)	χ2 = 14.57
Primary (1–8)	19(20.7)	50(27.3)	df = 2
Secondary and above	5(5.4)	37(20.2)	*p*-value = 0.001
Husband educational level			
Illiterate	49(55.7)	62(36.9)	χ2 = 8.91
Primary (1–8)	33(37.5)	83(49.4)	df = 2
Secondary and above	6 (6.8)	23(13.7)	*p*-value = 0.012
Maternal occupation			
Housewife	40(43.5)	89(48.6)	χ2 = 2.772
Farmer	44 (47.8)	70 (38.3)	df = 3
Employed	6 (6.5)	17 (9.3)	*p*-value = 0.428
Student	2(2.2)	7 (3.8)	
Ethnicity			
Tigre	90 (97.8)	177 (96.7)	χ2 = 0.265, df = 1
Amhara	2(2.2)	6(3.3)	*p*-value = 0.607
Religion of respondents			
Orthodox	92(100)	182 (99.5)	χ2 = .505, df = 1
Muslim	0(0.0)	1 (0.5)	*p*-value = 0.478
Have a radio or TV			
No	69 (75)	83 (45.4)	χ2 = 21.76, df = 1
Yes	23(25)	100 (54.6)	*p*-value = 0.000

^a^Single, widowed and divorced

### Reproductive and obstetric related characteristics of study participants

The majority of cases (85, 92.4%) and 136 (74.3%) controls had parity of 2+ and 13 (14.1%) cases and 25 (13.7%) controls had an abortion in their lifetime. Sixteen (17.4%) cases and 27 (14.8%) controls ever had a stillbirth. Nearly two-thirds (64.1%) of cases and the majority controls 170 (92.9%) had ANC visits during their last pregnancy. Out of these, 32 (54.2%) and of 90(52.9%) started ANC follow up at 12–24 weeks of gestation, Sixty-seven (72.8%) cases had “poor knowledge” of danger signs during pregnancy, but 108 (59%) controls had “good knowledge” of danger signs. There was a significant difference between cases and controls in terms of parity, a number of ANC visits, knowledge of danger signs during pregnancy and knowledge of obstetric complications (Table 2).

**Table 2:** Reproductive and obstetric related characteristics of study participants in Tanqua-Abergele District, Tigray, north Ethiopia, 2014

Variables	Cases No (%)	Controls No (%)	Chi-square, df, *p*-value
Parity			
Primipara	7(7.6)	47(25.7)	χ2 = 12.674, df =1
Multiparous	85(92.4)	136(74.3)	*P*-value = 0.000
Ever had abortion			
No	79(85.9)	158(86.3)	χ2 = 0.011, df =1
Yes	13(14.1)	25(13.7)	*P*-value = 0.915
Number of abortion			
1	10(76.9)	17(68.0)	χ2 = 0.331, df =1
> =2	3(23.1)	8(32.0)	*P*-value = 0.565
Ever had still birth			
No	76(82.6)	156(85.2)	χ2 = 0.323, df =1
Yes	16(17.4)	27(14.8)	*P*-value = 0.570
Number of still birth			
1	10(62.5)	21(77.8)	χ2 = 1.166, df =1
> =2	6(37.5)	6(22.2)	*P*-value = 0.280
Last pregnancy planned			
No	20(21.7)	29(15.8)	χ2 = 1.411, df =1
Yes	72(78.3)	154(84.2)	*P*-value = 0.228
Number of ANC visit			
no visit	33(35.9)	13(7.1)	χ2 = 62.594
1–3visit	45(48.9)	59(32.2)	df = 2
four and above	14(15.2)	111(60.7)	*p*-value = 0.000
Timing of ANC follow-up			
< 12 weeks	20(33.9)	71(41.8)	χ2 = 3.402
12–24weeks	32(54.2)	90(52.9)	df = 2
> 24 weeks	7(11.9)	9(5.3)	*p*-value = 0.183
Ever had obstetric complication			
No	60(65.2)	115(62.8)	χ2 = 0.149, df =1
Yes	32(34.8)	68(37.2)	*P*-value = 0.699
Decision maker for place of birth			
Non autonomous	28(30.5)	45(24.6)	χ2 = 1.073, df =1
Autonomous	64(69.5)	138(75.4)	*P*-value = 0.300
knowledge on danger signs of pregnancy			
Poor knowledge	67(72.8)	75(41)	χ2 = 24.857, df =1
Good knowledge	25(27.2)	108(59)	*P*-value = 0.000
General knowledge of obstetric complication			
Poor	82(89.1)	99(54.1)	χ2 = 33.475
Fair	5(5.4)	36(19.7)	df = 2
Good	5(5.4)	48(26.2)	*p*-value = 0.000

**Fig. 1:**
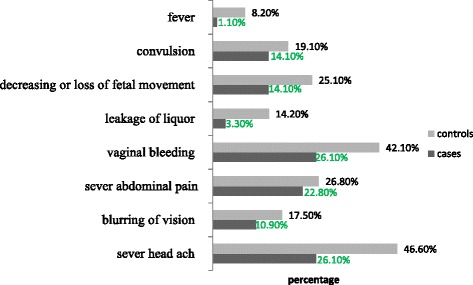
Percentage of women’s knowledge on danger sign during pregnancy in Tanqua-Abergele district, Central zone, Tigray, Ethiopia, 2014

**Fig. 2:**
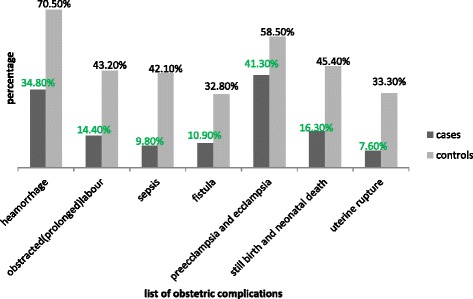
Percentage of women’s knowledge on general obstetric complications in Tanqua-Abergele district, Central zone, Tigray, Ethiopia, 2014

Most commonly mentioned danger signs during pregnancy by cases were vaginal bleeding (26.1%), severe headache (26.1%) and severe abdominal pain (22.8%). Controls cited a severe headache (46.6%), vaginal bleeding (42.1%) and severe abdominal pain (26.8%) as danger signs (Fig. 1). Regarding knowledge of obstetric complications, the three most common complications revealed by cases and controls were hemorrhage(34.8% and 70.5%), preeclampsia and eclampsia (41.3% and 58.5%), and stillbirth and neonatal death (16.3% and 45.4%), respectively (Fig. 2).

### Healthcare infrastructure and transportation

Median times to travel to the nearest health facility (health center) for cases and controls were 90 (IQR ± 90) and 60 (IQR ± 100) minutes, respectively. Concerning mode of transport, 48 (45.2%) cases and 74 (40.4%) of controls cited walking. There was a significant difference between cases and controls in the availability of health centers, time to reach the nearest health institution, preference of ambulance service, and ever having received counseling about the place of delivery (Table 3).

**Table 3:** Health care (Programme) related characteristics of study participants in Tanqua-Abergele District, Tigray, north Ethiopia, 2014

Variables	Cases No (%)	Controls No (%)	Chi-square, df, *p*-value
Presence of health facility in their kebelle			
No	40(43.5)	46(25.1)	χ2 = 9.583, df =1
yes	52(56.5)	137(74.9)	*P*-value = 0.002
Road accessibility to the HC			
no	33(35.9)	31(16.9)	χ2 = 12.286, df = 1
yes	59(64.1)	152(83.1)	*P*-value = 0.000
Mode of transportation			
Ambulance	45 (45.7)	103(56.3)	χ2 = 3.47
Public transport	2(2.2)	6(3.3)	df =2
On foot	48(45.2)	74(40.4)	*P*-value = 0.176
Time to reach the HC in minutes			
< 60 min	27(29.3)	111(60.7)	χ2 = 24.738
60–120 min	39(42.4)	48(26.2)	df =2
> 120 min	26(28.3)	24(13.1)	*P*-value = 0.00
Preferred getting ambulance service			
no	22(23.9)	23(12.6)	χ2 = 5.757, df =2
yes	70(76.1)	160(87.4)	*P*-value = 0.016
Ever gotten counselling about place of delivery			
No	10(10.9)	2 (1.1)	χ2 = 14.022, df = 1
yes	82(89.1)	181(98.9)	*P*-value = 0.000
Counselling provider (*n* = 263)			
health workers	34(41.5)	81(44.8)	χ2 = 18.252
women development army(WDA)	6(7.3)	28(15.5)	df =4
Family(husband, Mother, father)	18(22.0)	11(6.1)	*P*-value = 0.001
health worker and WDA	14(17.1)	25(13.8)	
health worker WDA and family	10(12.2)	36(19.9)	

### Factors associated with home delivery

Adjusting for other variables, women who did not possess a radio or television had increased odds of home delivery compared to their counterparts (AOR: 7.24, 95% CI: 2.71–19.33). Similarly, women who did not have an ANC follow-up (AOR: 10.41, 95% CI: 2.92–37.19) and those with1-3 ANC visits only (AOR: 4.75, 95% CI: 1.69–13.31) had greater odds of delivering at home compared with women who had 4 or more ANC visits. The odds of home delivery was 8.75 times greater among women with “poor knowledge”of obstetric complications as compared to women who had “good knowledge”(AOR: 8.75, 95% CI: 2.32–32.92). Time to reach health institution on foot was strongly associated with home delivery. The odds of home delivery was5.15 times higher among women living more than two hours walking distance to the nearest health center compared to those within one hour of the nearest center (AOR: 5.15, 95% CI: 1.28–20.70) (Table 4).

**Table 4:** Predictors of Home Delivery in Tanqua-Abergele District, Tigray, north Ethiopia, 2014

Variables	Cases No (%)	Controls No (%)	Crude OR (95% CI)	Adjusted OR (95% CI)
Residence				
Rural	90(97.8)	138 (75.4)	14.67(3.47–61.99)	2.83(0.42–19.17)
Semi rural	2 (2.2)	45(24.6)	1	1
Have a radio or TV				
No	69 (75)	83 (45.4)	3.61(2.08–6.29)	7.24(2.71–19.33)^a^
Yes	23(25)	100 (54. 6)	1	1
Have a radio or TV				
No visit at all	33(35.9)	13(7.1)	20.13 (8.61–47.04)	10.41(2.92–37.19)^a^
1–3 visits	45(48.9)	59(32.2)	6.05 (3.07–11.91)	4.75(1.69–13.31)^a^
Four and above	14(15.2)	111(60.7)	1	1
Knowledge on danger signs of pregnancy				
Poor	67(72.8)	75(41)	3.86(2.24–6.66)	1.75(0.65–4.75)
Good	25(27.2)	108(59)	1	1
Knowledge of obstetric complication				
Poor	82(89.1)	99(54.1)	7.95 (3.03–20.90)	8.75(2.32–32.92)^a^
Moderate	5(5.4)	36(19.7)	1.33 (0.36–4.96)	1.97(0.33–11.83)
Good	5(5.4)	48(26.2)	1	1
Time to reach the HC in minutes				
< 60 min	27(29.3)	111(60.7)	1	1
60–120 min	39(42.4)	48(26.2)	3.34 (1.84–6.06)	1.46(0.52–4.14)
> 120 min	26(28.3)	24(13.1)	4.45(2.22–8.94)	5.15(1.28–20.70)^a^
Presence of health facility in the kebele				
No	40(43.5)	46(25.1)	2.29(1.35–3.89)	0.60(0.18–1.95)
Yes	52(56.5)	137(74.9)	1	1
Road accessibility to the HC				
No	33(35.9)	31(16.9)	2.74(1.54–4.87)	0.99(0.35–2.83)
Yes	59(64.1)	152(83.1)	1	1

^a^Significantly associated at *P* < 0.05

## Discussion

In Ethiopia, 88.3% of births are delivered at home [15]. This high rate of home delivery is explained by several factors related to education, wealth, and access to healthcare. In our study, women who didn’t possess a radio or television, those who didn’t attend ANC at all or had few visits, those with poor knowledge of obstetric complications, and those living far away from health institutions all had higher odds of home delivery.

Our finding showing increased odds of institutional delivery among women owning a radio or television is consistent with other studies in Ethiopia, Ghana, Indonesia, and India [15–19]. Radio and television ownership likely increases women’s knowledge about pregnancy and labor, including danger signs and complications, and perhaps contributes to advance planning.

ANC is the most favorable contact point for mothers to get more information about the risks and problems they may encounter during delivery. The World Health Organization (WHO) recommends that women without complications should have at least four antenatal visits, the first of which should take place during the first trimester [15]. Our study revealed a strong association between fewer ANC visits and home delivery. Studies from Kathmandu, Nepal, and Malawi showed a strong connection between no/fewer than four ANC visits and home delivery [9, 20]. Studies in Ghana’s well as North Showa, Dabat, East Wollega, and Sekela District and Munisa Districts in Ethiopia showed that women who had more prenatal visits were more likely to deliver at the health institutions [16, 17, 19, 21–23] Failing to capitalize on ANC visits could be significant missed opportunity in the FMoH’s efforts to reduce maternal mortality and morbidity.

Knowledge of obstetric complications was a strong predictor of home delivery in our study. The odds of home delivery were considerably higher among women with “poor knowledge “of obstetric complications. This result was consistent with a study in Bahir Dar [11]. Women with advanced knowledge of potential complications and danger signs are better able to choose preferred delivery location and attendant as well as arrange for transport to skilled care in case of emergency. Women are also able to arrange companions to travel with them.

Our study showed a significant association between walking distance to the nearest health facility and home delivery. Women who lived greater than two hours walking time (distance) to the nearest health center as compared to those less than one hour walking time had considerably higher odds of delivering at home. This finding is in line with studies in Kathmandu, Nchelenge and Kaoma, Zambia, and East Wollega, Ethiopia [9, 10, 21, 24, 25]. Women facing long walks may be particularly unwilling to consider travel to health centers during the critical hours preceding delivery. In a country where 85% of the population resides in rural areas with poor infrastructure and inaccessible roads, many Ethiopian women are simply too far from a facility to consider institutional delivery. Furthermore, where transportation is available, it may not be suitable for laboring women.

### Limitations

Our study includes several limitations: The sampling frame was constrained to the records of mothers who gave birth at a health institution or home as contained in the Tanqua-Abergele family folder, which may not be comprehensive. Also, our study didn’t collect information from the perspective of healthcare providers and husband to substantiate our findings.

## Conclusion

Unable to meet the minimum requirement WHO of ANC service had a potential to give birth at home. Investing in infrastructure will contribute to improving maternal health. Having a different source of media (radio or television) could have a role in increasing the institutional delivery. Policy makers and other concerned bodies should give due attention to the fulfillment of infrastructure and educate women on the importance of institutional delivery.

## Abbreviations

ANC: Antenatal care
AOR: Adjusted odd ratio
CI: Confidence interval
IEOS: Integrated emergency obstetric and surgery
SPSS: Statistical package for social science
SSA: Sub-Sharan Africa
WHO: World Health Organization
